# Supplementary material for: Impact of Iron on the Fe–Co–Ni Ternary Nanocomposites Structural and Magnetic Features Obtained via Chemical Precipitation Followed by Reduction Process for Various Magnetically Coupled Devices Applications
Source: Nanomaterials (Basel). 2021 Jan 29;11(2):341. doi: 10.3390/nano11020341 (PMC7910875; doi:10.3390/nano11020341)
Supplement: Supplementary file 1 [file nanomaterials-11-00341-s001.pdf]

Supplementary Material

Impact of Iron on the Fe–Co–Ni Ternary Nanocomposites Structural and Magnetic Features Obtained *via* Chemical Precipitation Followed by Reduction Process for Various Magnetically Coupled Devices Applications

Tien Hiep Nguyen [1,2], Gopalu Karunakaran [3,*], Yu.V. Konyukhov [1,*], Nguyen Van Minh [4,5,*], D.Yu. Karpenkov [6] and I.N. Burmistrov [7,1]

[1] Department of Functional Nanosystems and High-Temperature Materials, National University of Science and Technology "MISiS", 119049 Moscow, Russia; htnru7@yandex.ru (T.H.N.)
[2] Department of Materials Science and Engineering, Le Quy Don Technical University, Hanoi 100000, Vietnam
[3] Biosensor Research Institute, Department of Fine Chemistry, Seoul National University of Science and Technology (Seoul Tech), Seoul 01811, Korea
[4] Institute of Research and Development, Duy Tan University, Danang 550000, Vietnam
[5] Institute of Technology, Hanoi 100000, Vietnam
[6] Faculty of Physics, Lomonosov Moscow State University, GSP-1, Leninskie Gory, 119991 Moscow, Russia; karpenkov_d_y@mail.ru (D.Y.K.)
[7] Engineering Centre, Plekhanov Russian University of Economics, Moscow 117997, Russia; glas100@yandex.ru (I.N.B.)
* Correspondence: karunakarang5@seoultech.ac.kr (G.K.); martensit@mail.ru (Y.V.K.); nguyenvanminh15@duytan.edu.vn (N.V.M.)

Table S1. Results of the TG analysis of obtained hydroxide precipitates under hydrogen flow.

Samples	ΔT, °C	First step			Second step			Third step			Fourth step			Fifth step		
		ΔT_1,°C	Tm_1, °C	$Vm_1 \cdot 10^5, s^{-1}$	ΔT_2,°C	Tm_2, °C	$Vm_2 \cdot 10^5, s^{-1}$	ΔT_3,°C	Tm_3, °C	$Vm_3 \cdot 10^5, s^{-1}$	ΔT_4,°C	Tm_4, °C	$Vm_4 \cdot 10^5, s^{-1}$	ΔT_5,°C	Tm_5, °C	$Vm_5 \cdot 10^5, s^{-1}$
1Fe–Co–Ni	25-700	25-130	121	22.9	130-210	181	25.2	210-300	257	41.4	300-360	348	63.6	360-450	378	48.2
3Fe–Co–Ni	25-700	25-130	112	10	130-200	152	9.2	200-310	258	24.2	310-390	362	30.9	390-550	467	39.5
5Fe–Co–Ni	25-700	25-130	108	21.7	130-200	154	16.5	200-320	260	22.4	320-395	365	29.8	395-550	470	35.3

Annotation. ΔT – the overall process temperature range, °C; ΔT_1, ΔT_2, ΔT_3, ΔT_4, ΔT_5 – temperature ranges of the steps, °C; Tm_1, Tm_2, Tm_3, Tm_4, Tm_5 – temperatures of maximum speed of the steps, °C; Vm_1, Vm_2, Vm_3, Vm_4, Vm_5 – maximum specific speeds of the steps, s^{-1}.

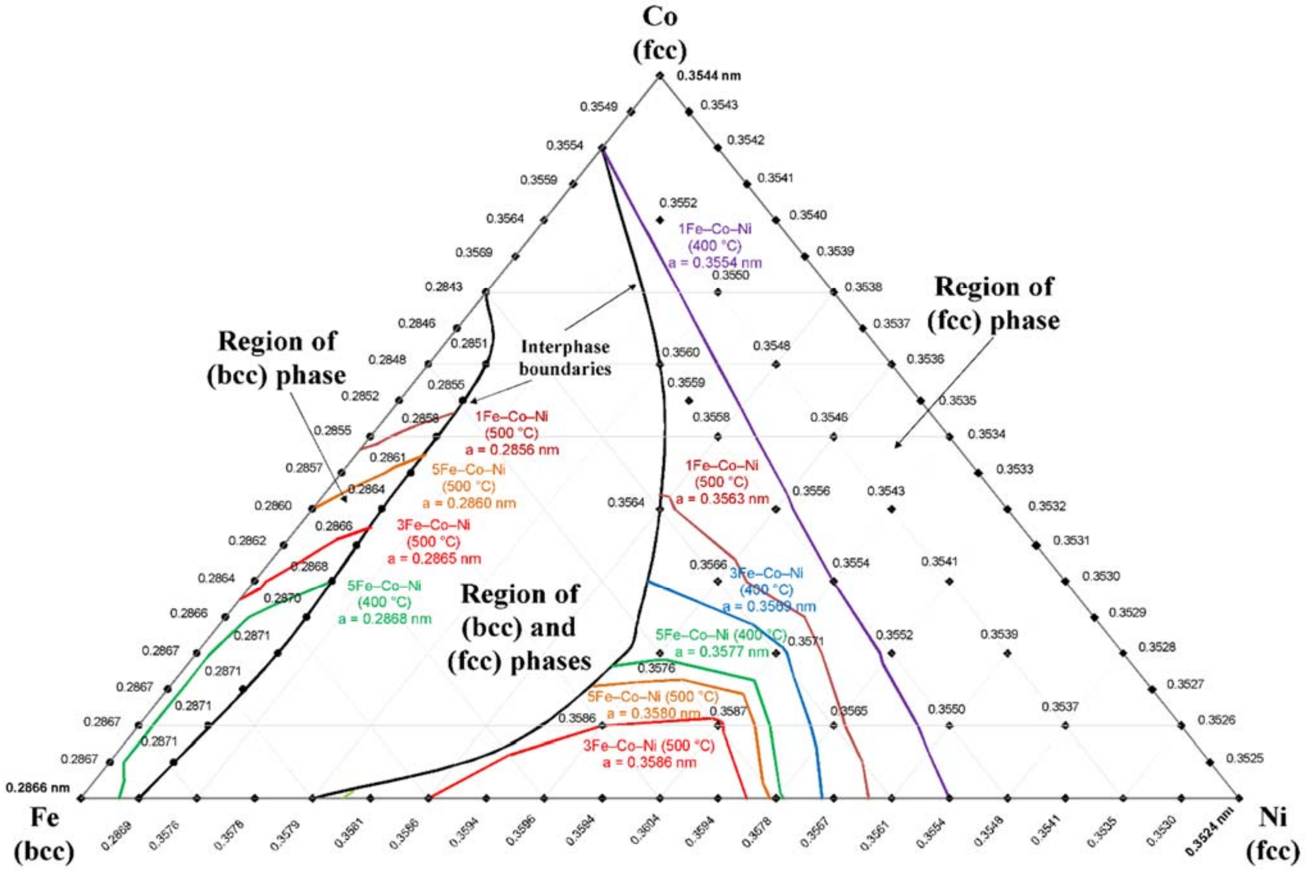

Figure S1. Triple Fe–Co–Ni state diagram (T = 500 °C).

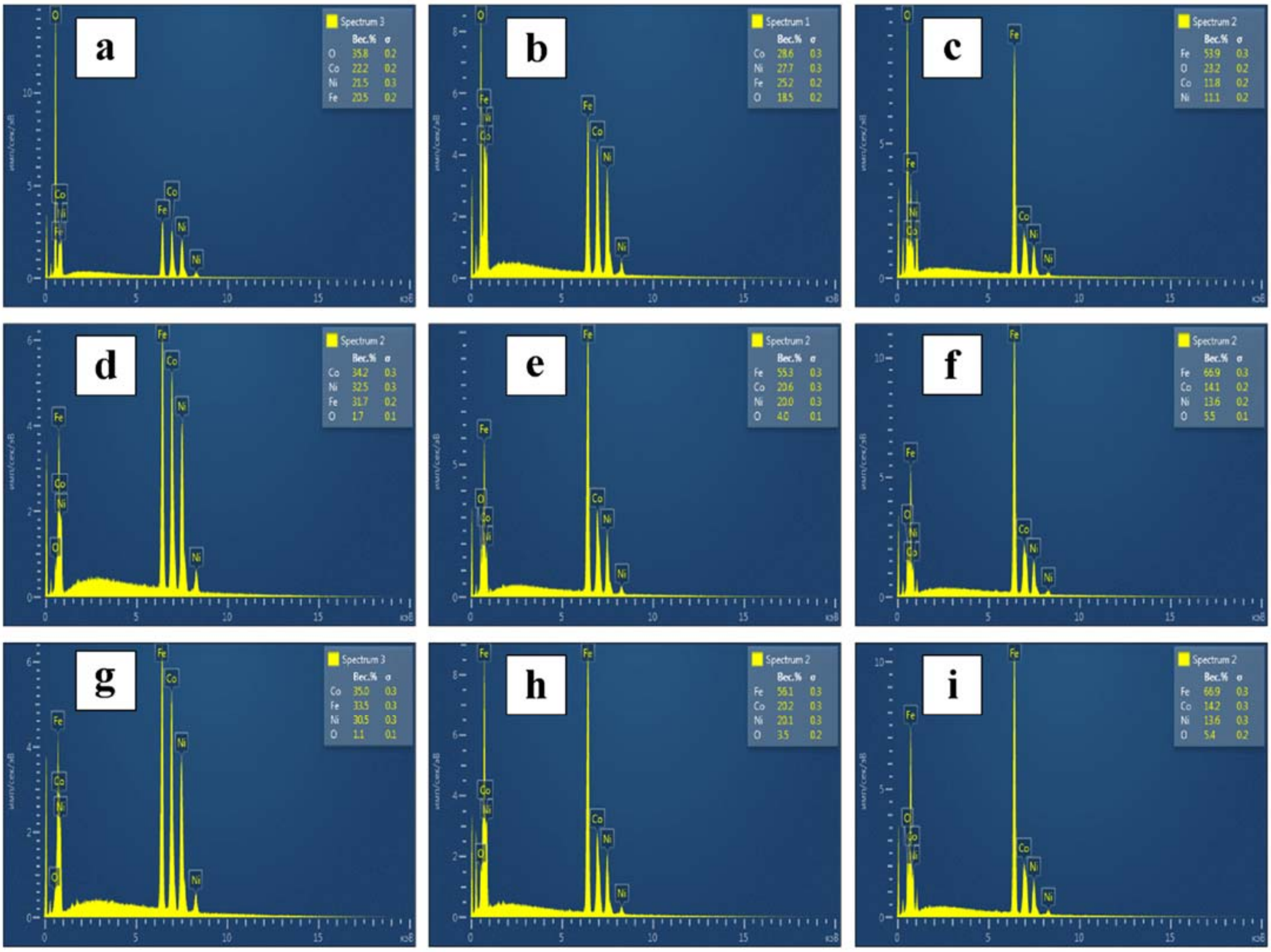

Figure S2. Results of EDX analysis. (**a**) 1Fe–Co–Ni T_R = 300, (**b**) 3Fe–Co–Ni T_R = 300, (**c**) 5Fe–Co–Ni T_R = 300, (**d**) 1Fe–Co–Ni T_R = 400, (**e**) 3Fe–Co–Ni T_R = 400, (**f**) 5Fe–Co–Ni T_R = 400, (**g**) 1Fe–Co–Ni T_R = 500, (**h**) 3Fe–Co–Ni T_R = 500, (**i**) 5Fe–Co–Ni T_R 500; T_R – reduction temperature, °C.

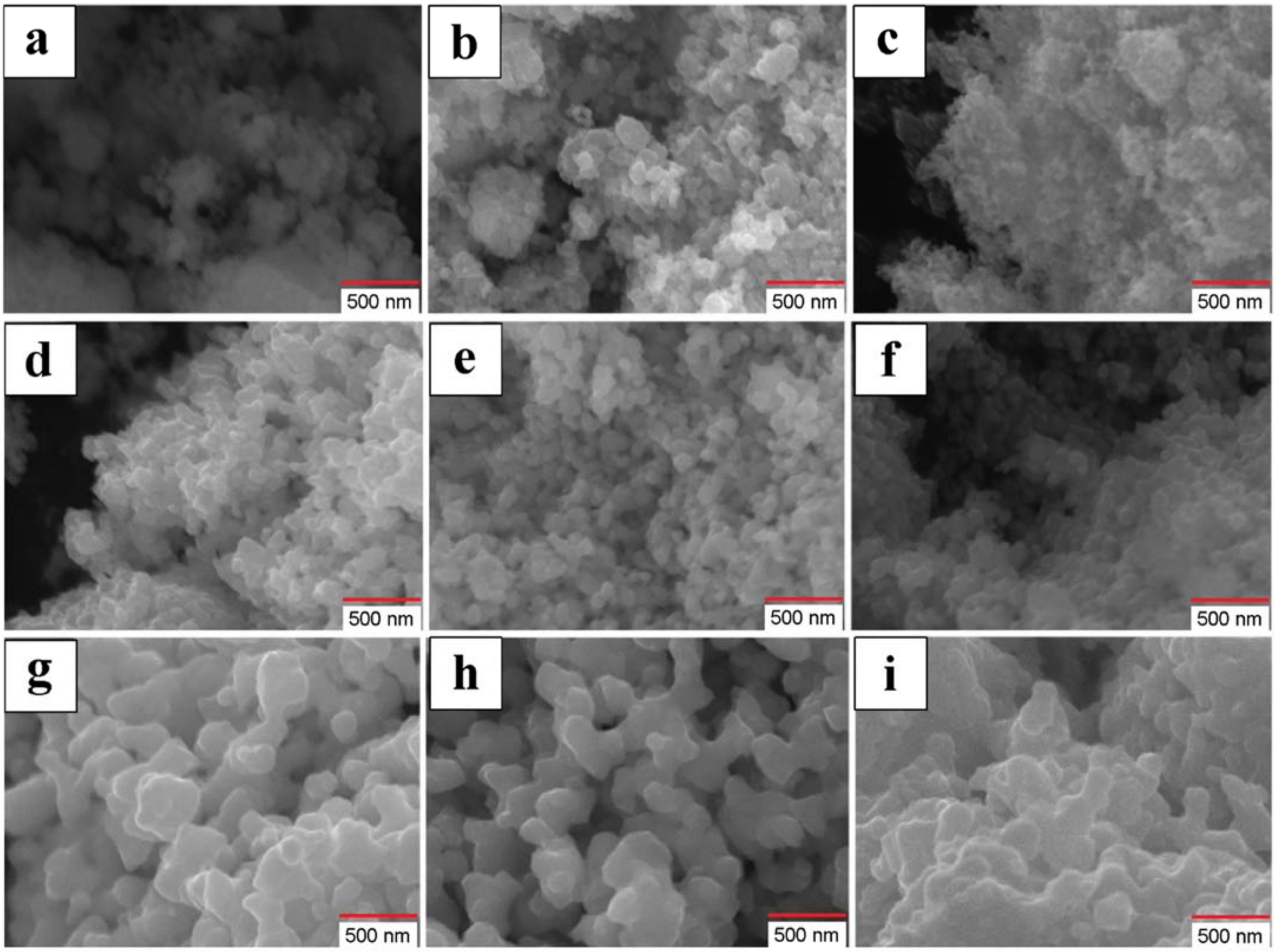

Figure S3. The SEM images of investigated. (**a**) 1Fe–Co–Ni $T_R = 300$, (**b**) 3Fe–Co–Ni $T_R = 300$, (**c**) 5Fe–Co–Ni $T_R = 300$, (**d**) 1Fe–Co–Ni $T_R = 400$, (**e**) 3Fe–Co–Ni $T_R = 400$, (**f**) 5Fe–Co–Ni $T_R = 400$, (**g**) 1Fe–Co–Ni $T_R = 500$, (**h**) 3Fe–Co–Ni $T_R = 500$, (**i**) 5Fe–Co–Ni $T_R = 500$, T_R – reduction temperature, °C.

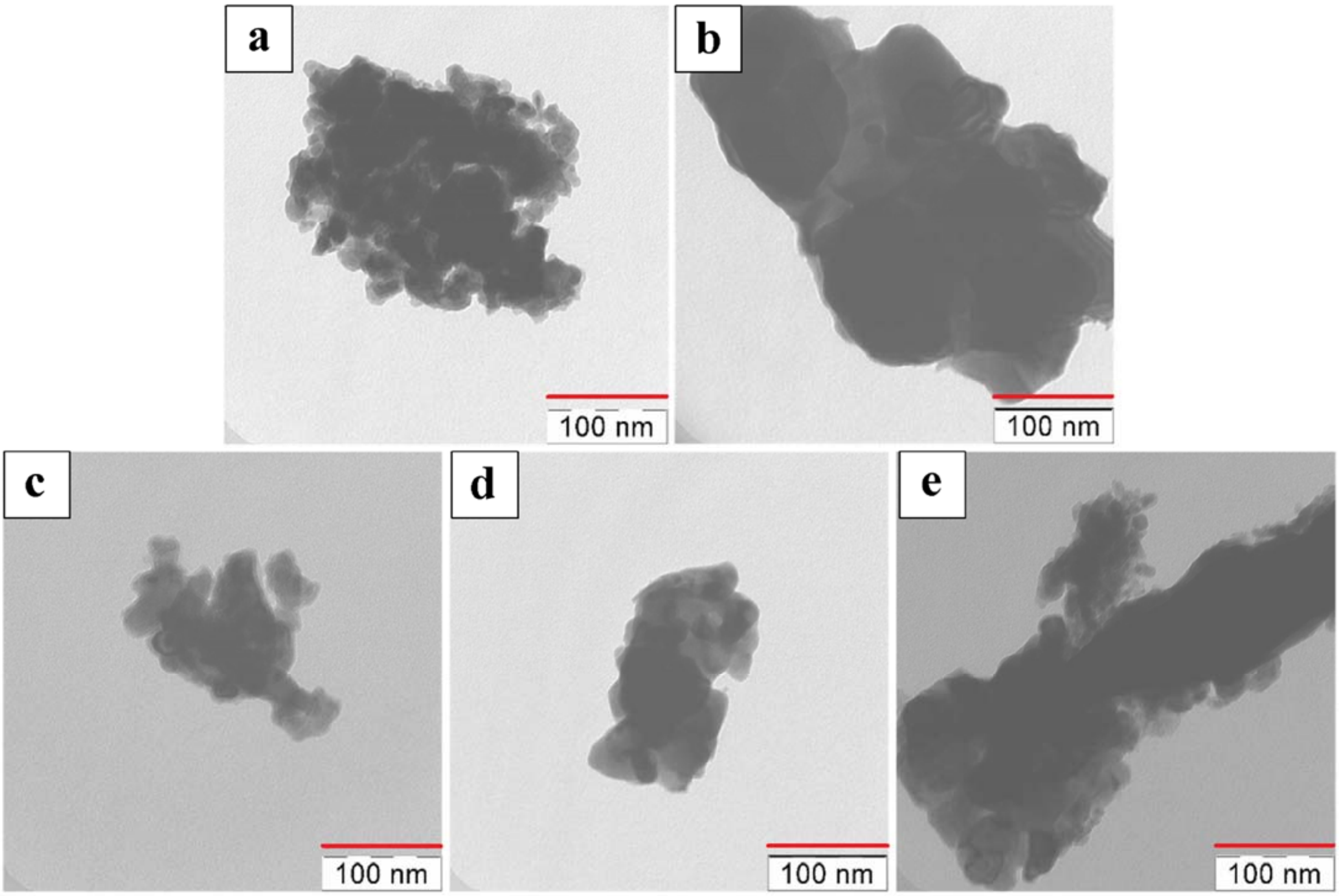

Figure S4. The TEM images of investigated samples. (**a**) 1Fe–Co–Ni $T_R = 300$, (**b**) 1Fe–Co–Ni $T_R = 500$, (**c**) 1Fe–Co–Ni $T_R = 400$, (**d**) 3Fe–Co–Ni $T_R = 400$, (**e**) 5Fe–Co–Ni $T_R = 400$, T_R – reduction temperature, °C.

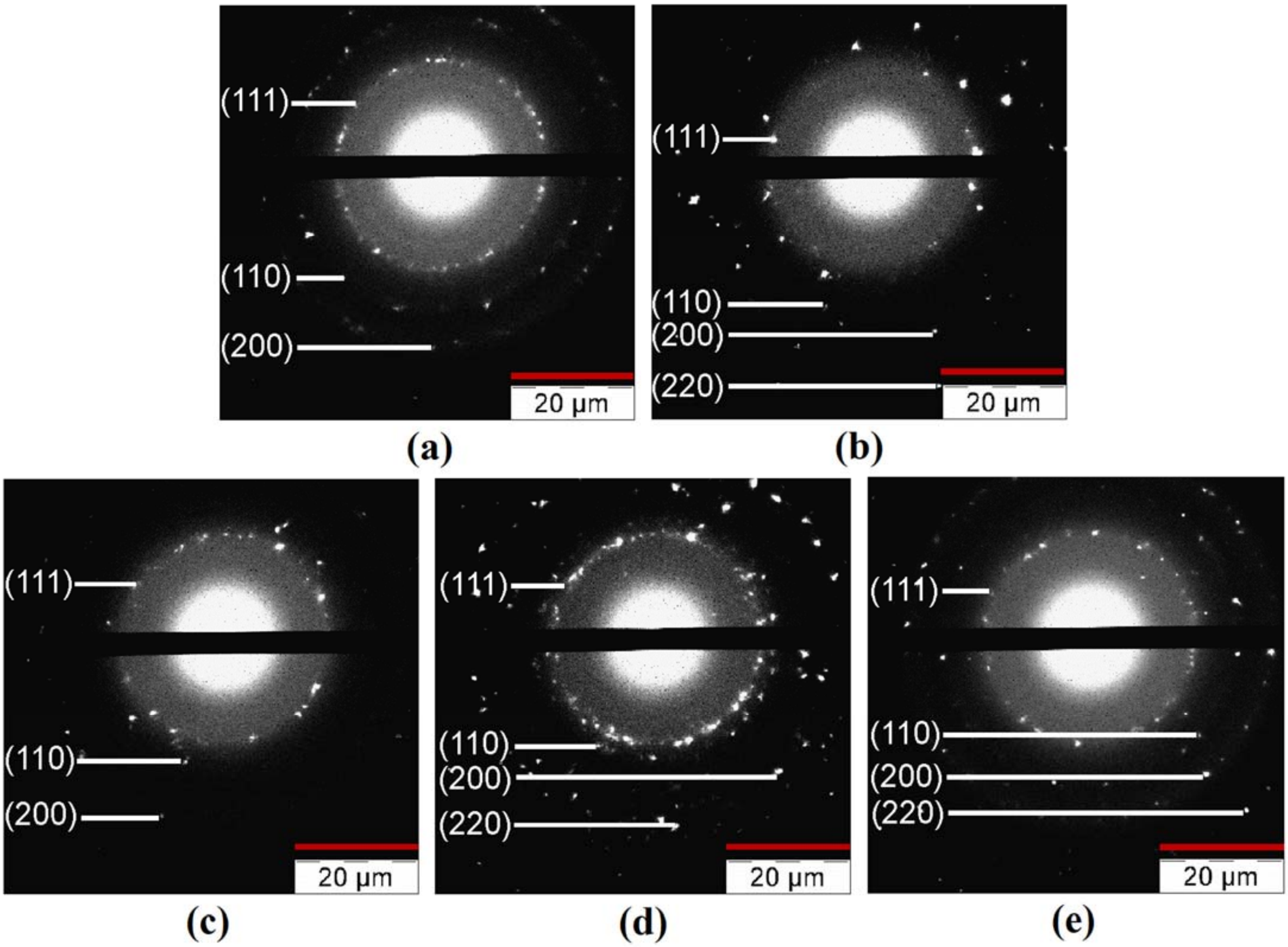

Figure S5. SAED of investigated samples. (**a**) 1Fe–Co–Ni $T_R = 300$, (**b**) 1Fe–Co–Ni $T_R = 500$, (**c**) 1Fe–Co–Ni $T_R = 400$, (**d**) 3Fe–Co–Ni $T_R = 400$, (**e**) 5Fe–Co–Ni $T_R = 400$, T_R – reduction temperature, °C.
